# Effectiveness of National Residential Smoking Cessation Program

**DOI:** 10.3390/ijerph18189901

**Published:** 2021-09-20

**Authors:** Mi-Jeong Park, Young-Gyun Seo, Hye-Mi Noh, Yeol Kim, Jong Lull Yoon, Yu-Jin Paek

**Affiliations:** 1 Department of Family Medicine, Hallym University Dongtan Sacred Heart Hospital, Hwaseong 18450, Korea; bonamj74@gmail.com (M.-J.P.); lull@hallym.ac.kr (J.L.Y.); 2 Department of Family Medicine, Hallym University Sacred Heart Hospital, Anyang 14068, Korea; yg035@daum.net (Y.-G.S.); noham111@hanmail.net (H.-M.N.); 3 National Cancer Center, Division of Center for Cancer Prevention and Detection, Goyang 10408, Korea; drheat@ncc.re.kr; 4 National Cancer Center, National Cancer Control Institute, Goyang 10408, Korea

**Keywords:** COVID-19, tobacco, smoking, abstinence, residential smoking cessation program, varenicline, nicotine replacement therapy, chronic morbidity, nicotine dependence, self-efficacy

## Abstract

We aimed to investigate the effectiveness of the Korean national five-day residential smoking cessation program and the factors affecting the long-term smoking cessation of participants. The residential smoking cessation program (2017–2018) recruited smokers with a smoking duration ≥ 20 years and who have attempted to quit smoking more than twice and/or smokers with chronic morbidities. Participants underwent an intensive intervention, including individual psychological therapy, group therapy, medical counseling, and pharmacotherapy. The 6-month continuous abstinence rate (CAR) was assessed via self-reports, the urine cotinine levels, and/or expired-air carbon monoxide levels. Logistic regression was used to analyze the adjusted odds ratio (aOR) to assess factors related to smoking cessation. Overall, 484 participants who completed the residential program and questionnaire were evaluated. The 3- and 6-month CAR were 81.82% and 63.22%, respectively. The aOR of 6-month continuous abstinence was lower among participants with severe nicotine dependence (aOR: 0.46, 95% confidence interval [CI]: 0.26–0.81) and higher among participants with combination therapy of varenicline with short-term nicotine replacement therapy (NRT) (aOR: 1.64, 95% CI: 1.07–2.51), with higher self-efficacy (aOR: 1.97, 95% CI: 1.15–3.37). The residential smoking cessation program was effective. High self-efficacy, combination therapy of varenicline with short-term NRT, and low nicotine dependence were associated with a high 6-month CAR.

## 1. Introduction

The coronavirus disease (COVID-19) pandemic and the global tobacco epidemic are coexisting. Smoking is associated with the progression of COVID-19 [1]. Tobacco use may increase the risk of infection, disease severity, and poor disease outcomes from COVID-19 [2]. Traditionally, tobacco smoking is a major cause of cardiovascular disease, respiratory disease, and cancer [3], and smokers have a 2–3 times higher mortality rate than never-smokers [4]. In 2015, 24.9% of the worldwide population aged 15 years and older were current smokers [5]. Quitting smoking can reduce the risk of smoking-related disease and premature death [6]. However, smoking cessation is challenging because of nicotine dependence, and smoking is considered as a chronic, relapsing medical condition that requires long-term management. Annually, only 3–5% of untreated smokers successfully quit smoking [7]. Smoking cessation interventions, including physician advice, counseling, and pharmacotherapy, can further increase the rate of smoking abstinence [8,9,10]. However, a systematic review revealed that smoking cessation for 6 months showed a low efficacy of 20–30% to maintain smoking abstinence [11].

As a more intensive intervention for smoking cessation, the Mayo Clinic developed an 8-day residential intensive treatment program that has been implemented since 1992 [12]. The program involves intensive behavioral and pharmacological treatment for smokers who have severe tobacco dependence and has shown higher rates of 6-month smoking abstinence (52%) than outpatient treatment (27%) [13].

In South Korea, the prevalence of smoking among adults (≥19 years) has decreased to 22.3% in 2017 from 35.1% in 1998 [14]. However, the smoking rate in male adults (≥19 years) remains remarkably higher at 38.1% [14] than that the average of 23% in other member countries of the Organization for Economic Cooperation and Development [15]. Accordingly, the Korean government has enforced tobacco control policies and increased tobacco tax in 2015. Additionally, Tobacco Control Centers were established in over 18 regions of the country. These centers target those with difficulty receiving conventional smoking cessation support services, including women, persons with disabilities, employees of small-sized enterprises, teenagers not under school supervision, and college students. In addition, they also provide residential smoking cessation programs (smoking cessation camp) for smokers. The Korean smoking cessation camp program is a government-funded initiative that was developed by benchmarking the Mayo Clinic 8-day residential intensive treatment program. Residents can participate in the camp for free. Smoking cessation medications (e.g., varenicline, bupropion, or nicotine replacement therapy (NRT)) may also be provided free of charge for up to 9 months. A multidisciplinary team consisting of physicians, nurses, and counselors provides a 5-day intervention and 6-month follow-up after discharge. As one of the regional tobacco control centers in Korea, we have operated the residential intensive smoking cessation program since 2015. In this study, we aimed to (1) investigate the rate of 6-month smoking abstinence and (2) determine the predictors of smoking cessation among smokers who participated in our program.

## 2. Materials and Methods

### 2.1. Study Design and Participants

The subjects were smokers who participated in a 5-day residential smoking cessation program between 1 January 2017 and 31 December 2018 at one of the regional tobacco control centers in the south of Seoul, Southern Gyeonggi Province. The inclusion criteria set by the National Tobacco Control Center of the Ministry of Health and Welfare of Korea were as follows: (1) subjects with smoking duration ≥20 years who have attempted to quit smoking more than twice and/or (2) subjects with chronic morbidities (i.e., hypertension, dyslipidemia, and diabetes). Of a total of 570 participants in a 5-day residential smoking cessation program between 1 January 2017 and 31 December 2018, 484 participants who completed the smoking cessation program and questionnaire were evaluated. We excluded 86 participants who did not complete the smoking cessation program and questionnaire.

This study was conducted in accordance with the Declaration of Helsinki. The study protocol was approved by the Institutional Review Board of Hallym University Sacred Heart Hospital (approval number: 2019-01-023). All subjects provided written informed consent before participation in the study.

### 2.2. Measurements

Data on demographic and socioeconomic characteristics of the subjects were collected. Information on age, sex, educational attainment, marital status, occupation, income, and method of recommendation were also collected. Alcohol consumption was classified as non-drinking or habitual drinking. Information on the diagnosis of hypertension, diabetes mellitus, and dyslipidemia by doctors was also collected. We also investigated whether the participants had a supporter who would encourage smoking cessation. Data on smoking-related history included the types of tobacco products, number of cigarettes smoked per day, duration of smoking, and results of the Fagerström Test for Nicotine Dependence (FTND). The psychiatric assessment included depressive symptoms; perceived stress; tobacco craving; Importance, Readiness, Confidence (IRC) for smoking cessation rulers; and self-efficacy scale for smoking cessation. Depressive symptoms were assessed using the Korean version of the Center for Epidemiologic Studies Depression (CES-D) Scale. Briefly, the CES-D is a reliable and validated 20-item self-report questionnaire [16] that has been used globally to screen for depression among adults. Each item is scored from 0 (rarely) to 3 (most or all of the time).

We divided the participants into three groups according to their overall scores as follows: severe depressive symptoms, scores ≥ 25; moderate depressive symptoms, scores ≥ 16–≤ 24; and mild and normal depressive symptoms, scores ≤ 15 [17]. Tobacco craving was assessed using the 47-item Tobacco Craving Questionnaire. IRC rulers have been established to be useful for predicting cessation [18]. Participants rated these three indexes on a scale of 1 to 10 (1 = not at all; 10 = most important, 100% ready, and 100% confident). Self-efficacy was measured using a Korean version of a validated 9-item self-efficacy/situational temptation scale constructed by Velicer, DiClemente, Rossi, and Prochaska [19] and translated by Kim [20]. The scale assesses a smoker’s self-efficacy, that is, the confidence to withstand various situations in which smokers are likely to smoke. The higher the score, the higher the confidence to withstand the temptation to smoke. When analyzing logistic regression, we categorized the self-efficacy scale into tertiles.

Health examinations, such as low-dose computed tomography, pulmonary function test, carotid ultrasonography, and blood and urine analysis, were performed.

### 2.3. Intervention

Standardized intervention for smoking cessation was provided by a multidisciplinary team at the regional tobacco control center. The multidisciplinary team involved physicians, nurses, and counselors who completed a smoking cessation specialist course. Candidates visited before starting the residential smoking cessation program. Physicians obtained a detailed medical history, performed a physical examination, and discussed smoking cessation medications (e.g., varenicline, bupropion, or NRT) with the participants, and developed an individualized medication plan. Counselors collected data on demographic, socioeconomic, and psychological factors using questionnaires. Table 1 shows the schedules of the residential smoking cessation program. The 5-day residential smoking cessation program included lectures on smoking cessation education (e.g., the harmfulness of tobacco, the mechanism and use of smoking cessation medications, dietary strategy and physical exercise after smoking cessation, and coping with smoking craving and stress) by professional instructors, daily check-up of vital signs and exhaled carbon monoxide (CO) by nurses, intensive psychological counseling by counselors (group counseling session and individual session), and counseling of the results of health-check up by physicians.

### 2.4. Follow-Up

Telephone or clinic visit follow-up was carried out at 2, 4, 6, 12, 18, and 24 weeks after completing the residential smoking cessation program. Structured interviews about coping with smoking craving, withdrawal symptoms, and methods of taking the smoking cessation medication were performed. Participants were advised to maintain smoking cessation medications for at least 3 months after program completion. Continuous abstinence was assessed via self-reports (telephone) and also biochemically via urine cotinine test (clinic visit) or expired-air CO level test (clinic visit). Urine cotinine was determined as positive or negative using an immunoassay dipstick method with cut-off 20 ng/mL (COT URINE RAPID TEST), and the cut-off of exhaled CO was 5 ppm. The results were recorded at each contact point. If the participants did not respond for more than 2 months, they were classified as smoking.

### 2.5. Statistical Analysis

The primary outcome measure was a 6-month continuous abstinence after completion of the residential smoking cessation program. The baseline characteristics are shown as the mean (standard deviation) or as the frequency (percentage). Between-groups differences were compared using the *t*-test, Mann–Whitney U test, paired *t*-test (for continuous variables), Fisher’s exact test, and the chi-squared test (for categorical variables). Multivariable logistic regression was performed to determine the adjusted odds ratio (OR) and 95% confidence interval (CI) to investigate factors associated with smoking cessation. We constructed three models for logistic regression. Model 1 was a series of univariable predictor models, model 2 tested each significant univariable predictor in a model with age and sex as covariates or moderators, and model 3 tested all significant univariable predictors and clinically significant factors in one model to determine which predictors accounted for unique variance. As a result, model 3 included age, sex, educational level, nicotine dependency (FTND score), types of pharmacotherapy, levels of depressive symptoms, and self-efficacy. All the tests were two-sided and were conducted using PASW Statistics version 21.0 (SPSS Inc., Chicago, IL, USA) and Stata/MP, version 14.0 (StataCorp, College Station, TX, USA). A *p*-value of <0.05 was considered statistically significant.

## 3. Results

**Figure 1:**
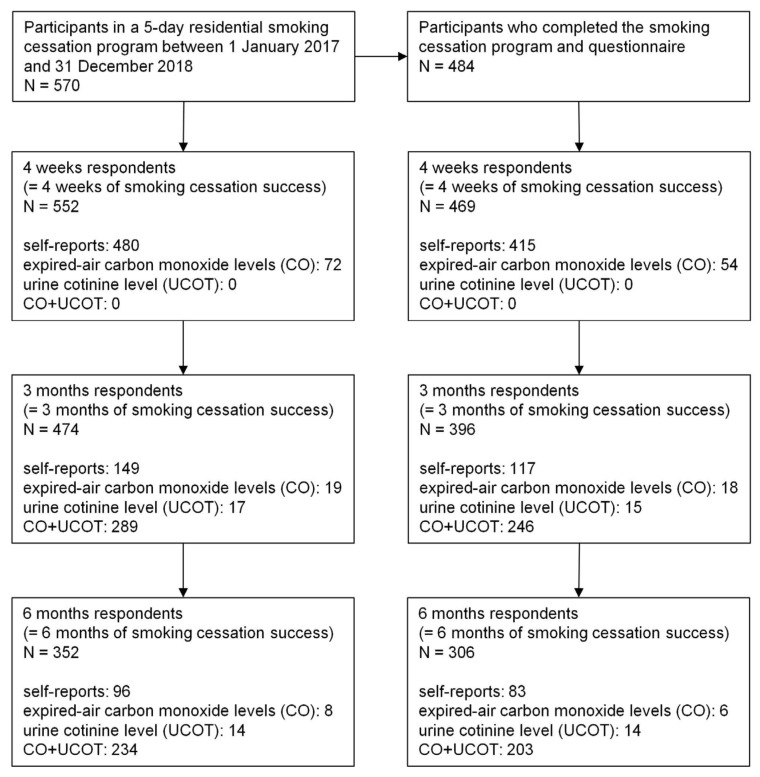
Participants flow chart.

**Figure 2:**
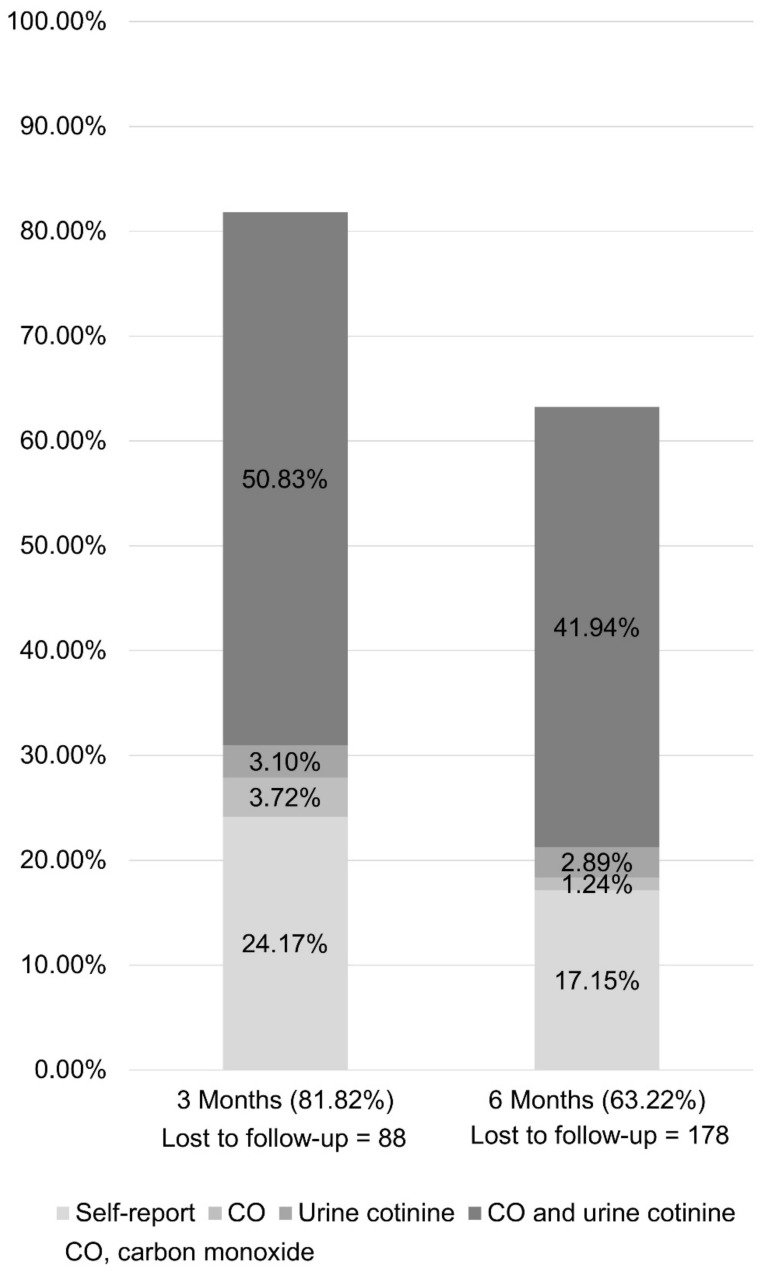
Abstinence rate by methods.

Figure 1 shows the flow chart of the participants in the study. The mean age was 55.32 ± 10.74, and 11.36% (n = 55) were female. The 4-week continuous abstinence rate (CAR) was 96.9%. The 3- and 6-month CAR was 81.82% and 63.22%, respectively. Meanwhile, the 3-and 6-month biochemically confirmed abstinence rate was 57.64% and 46.07%, respectively (Figure 2). From an intent-to-treat perspective, the 4-week, 3-month, and 6-month CAR was 96.84%, 83.16%, and 61.75%, respectively (Table 2).

Table 3 shows the general characteristics, smoking characteristics, and psychological characteristics of the study participants following the success of continuous abstinence at 4 weeks, 3 months, and 6 months. A comparison of success rates by sex showed that both the 3-month CAR (83.2% vs. 64.1%) and the 6-month CAR (70.9% vs. 56.4%) were higher in men than in women. Socioeconomic characteristics, comorbidities (hypertension, diabetes, dyslipidemia), and supporters for smoking cessation were not associated with CAR. Meanwhile, the FTND score was associated with the success rate of smoking cessation over the entire period. The FTND score was always significantly lower in the successful smoking group than that in the failed group. In total, 94.2% of all participants received NRT or combination therapy of varenicline with short-term NRT as a smoking cessation pharmacotherapy. Compared to the failure group, the proportion of participants who received combination therapy was significantly higher in the 4-week and 3-month success groups.

With respect to the baseline psychological assessment results, the 3-month success group had significantly higher confidence in smoking cessation, lower levels of depressive symptoms, lower craving for smoking, and higher self-efficacy than did the failed group. Meanwhile, the 6-month success group only had higher self-efficacy compared to the failed group. The average baseline self-efficacy score of the participants was 26.03 ± 6.72, and the self-efficacy score increased significantly to 34.10 ± 7.41 immediately after the program completion (*p*-value < 0.001). Table 4 and Table 5 show the results of logistic regression analysis of the data on factors associated with 3-month and 6-month smoking cessation, respectively. In logistic regression, pharmacotherapy was analyzed using the NRT group as a reference because the number of participants who did not receive any pharmacotherapy and those who received varenicline monotherapy was small. In the fully adjusted model (Table 4, Model 3), the odds of 3-month continuous abstinence was lower among participants with severe nicotine dependence (higher FTND score, OR: 0.28; 95% CI, 0.14–0.59) and those severe depressive symptoms (OR: 0.48; 95% CI: 0.25–0.91), whereas it was higher among participants who received combination therapy of varenicline and short-term NRT (OR: 2.13, 95% CI: 1.25–3.63). Further, in the fully adjusted model (Table 5, Model 3), the odds of 6-month continuous abstinence was lower among participants with moderate (OR: 0.46, 95% CI: 0.28–0.75) and those with severe (OR: 0.46, 95% CI: 0.26–0.81) nicotine dependence, whereas it was higher among participants who received combination therapy of varenicline and short-term NRT (OR: 1.64, 95% CI: 1.07–2.51), with higher self-efficacy (OR: 1.97, 95% CI: 1.15–3.37).

We also performed a logistic regression analysis excluding participants who did not receive any pharmacotherapy and those who received varenicline monotherapy. In the fully adjusted model (Table 6, Model 3), the odds of 3-month continuous abstinence was lower among participants with severe nicotine dependence (OR: 0.33; 95% CI, 0.15–0.71) and those severe depressive symptoms (OR: 0.44; 95% CI: 0.23–0.88), whereas it was higher among participants who received combination therapy of varenicline and short-term NRT (OR: 2.12, 95% CI: 1.24–3.61). Further, in the fully adjusted model (Table 6, Model 3), the odds of 6-month continuous abstinence was lower among participants with moderate (OR: 0.46, 95% CI: 0.28–0.76) and those with severe (OR: 0.52, 95% CI: 0.29–0.93) nicotine dependence, whereas it was higher among participants who received combination therapy of varenicline and short-term NRT (OR: 1.63, 95% CI: 1.07–2.49), with higher self-efficacy (OR: 1.75, 95% CI: 1.01–3.03).

## 4. Discussion

This analysis of participants in a Korean residential smoking cessation program found a high 6-month CAR of 63.2%. Further, lower nicotine dependency, combination therapy of varenicline with short-term NRT, and higher self-efficacy were found to be significantly associated with 6-month smoking cessation in smokers.

Several studies have reported the results of residential smoking cessation programs, most of which have a high 6-month success rate. In 2003, a 4-day residential smoking cessation program, including NRT, psychological group therapies, and education about relapse prevention skills, nutrition, and diet, reported a 26.1% success rate in the 7-day point abstinence rate at 6 months [21]. In 2011, Hays et al. reported the results of an 8-day residential smoking cessation program of the Mayo Clinic for 222 participants in comparison to outpatient treatment. The results showed a markedly higher self-reported 7-day point prevalence smoking abstinence rate at 6 months of the residential program than that of the outpatient intervention (52% vs. 27%) [13]. Ho et al. recently reported that a 3-day residential program in Hong Kong yielded a 57.5% self-reported 7-day point prevalence abstinence rate at 26 weeks [22]. In Korea, the 6-month self-reported CAR of the smoking cessation program for 954 outpatients was 30.5% [23]. We also found that the residential program was more effective than outpatient programs. In a systematic review of the smoking relapse curve, Hughes et al. reported that most relapses occurred early—within 8 days [7]. Therefore, intensive intervention is required in the first week of smoking abstinence. The residential programs can actively provide various treatments, including pharmacotherapies, behavioral therapy, and multiple activities, in the early stage of smoking cessation. In addition, participants can support each other, and this can help prevent early relapses. This study also showed remarkably high abstinence rates at 4 weeks (96.9%).

Nicotine is a determinant of tobacco addiction [24]. Further, nicotine dependence is known to be related to the success of smoking cessation [25]. Fagerström et al. reported that an increase of one unit in baseline FTND scores decreased the predicted CAR for week 24 by 2.5% for the varenicline trial group and only by 1.2% for the placebo control group [26]. Our study also found that the higher the FTND score, the lower the success rate, even after adjusting for other variables.

The results of the current showed that high self-efficacy was associated with a high 6-month CAR. This implies that although the residential program provided intensive intervention, baseline self-efficacy was also associated with long-term success rates. This is consistent with the findings of several previous studies that the higher the self-efficacy, the higher the success rate of smoking cessation [27,28]. Li et al. reported that smoking abstinence self-efficacy mediated the association between nicotine dependence and successful smoking cessation [29]. In a prospective cohort study of a Japanese smoking cessation clinic, Taniguchi et al. reported that the 12-week smoking cessation success group had markedly higher self-efficacy than the failed group [30]. These findings imply that focusing on interventions that strengthen self-efficacy will increase the success rate of smoking cessation. In this study, the result showed that the self-efficacy score increased after completion of the program. The residential program has the advantage of providing intensive psychological counseling and behavioral therapy compared to an outpatient clinic. Therefore, the residential program is expected to increase the abstinence rate of smoking by improving the self-efficacy of participants.

In general, smokers with high nicotine dependence are expected to have intense withdrawal symptoms [25]. Participants in the program were mainly smokers who had more than 20 years of smoking duration and attempted to quit smoking more than twice. Thus, they had various and intense withdrawal symptoms during the program. Accordingly, only a limited number of participants did not receive any medication to control withdrawal symptoms. Smokers who select varenicline monotherapy often need additional NRT to control withdrawal symptoms during the program. As such, the number of participants who received varenicline monotherapy was also small. In this study, combination therapy of varenicline with short-term NRT was more effective for successful smoking cessation than NRT monotherapy. In a meta-analysis of randomized controlled trials, combination therapy of varenicline with NRT was more effective than varenicline monotherapy, and there were no significant differences in adverse events between the two groups [31]. Based on these findings, combination therapy of varenicline and NRT could be an effective option for smokers who generally have a high smoking relapse rate.

Previous studies showed that education level, alcohol use, and supporters of smoking cessation were associated with the success of smoking cessation [25,32,33,34]. However, in this study, such factors were not related to the success of smoking cessation. The residential program is an intensive intervention that includes individual psychological therapy, group therapy, and pharmacotherapy, and thus it appears to have reduced the impact of other factors.

Only a small number of residential intervention programs for smoking cessation have been reported to date. This study is valuable as it shows the results of residential intervention programs for approximately 500 smokers and reaffirms that the residential programs are effective for smoking cessation. Further, unlike previous residential smoking cessation program studies that commonly used a 7-day point abstinence rate, we used a 6-month CAR as an outcome measure. Hughes et al. recommended using continuous or prolonged smoking abstinence to assess smoking cessation [35]. In addition, this study is valuable in that biochemically confirmed abstinence rates were also high at 3-month 57.64% and 46.07% at 6-month, unlike other studies that reported self-reported abstinence rates.

Although our study has several strengths, it also has some limitations. First, the participants were mainly smokers who have been smoking for more than 20 years. As such, we could not determine the effectiveness of the residential program for those with short smoking duration. Second, this study evaluated a 6-month success rate, but an additional analysis of the 1-year success rate is needed to assess the effectiveness of residential programs for long-term smoking cessation. In previous studies, the 1-year smoking cessation rate in outpatient smoking cessation clinics was approximately 30% [36,37]. Although direct comparisons are difficult because residential program participants may have a longer smoking history or may be more addicted than outpatient program participants, it is meaningful that residential program participants nevertheless had a higher smoking cessation rate. Third, this study is limited in that it is not a randomized controlled trial. However, compared to the 6-month CAR of smokers who enrolled in the Korean National Health Insurance Service smoking cessation outpatient program was 30.5% [23], it can be assumed that the 6-month CAR of the residential program is higher indirectly. Therefore, future studies, including smokers with short smoking duration, studies evaluating the longer-term effects of residential programs, and randomized controlled trials, are needed. Fourth, although the smoking cessation camp showed a high success rate of smoking cessation, a cost-effectiveness analysis was not performed. That is necessary because the cost per person is higher than that for outpatient clinics. Although not a residential program, Lee et al. reported that the smoking cessation programs for hospitalized patients are highly cost-effective interventions according to the recommended cost-effectiveness thresholds by the World Health Organization [38].

## 5. Conclusions

The Korean residential smoking cessation program showed a high 6-month continuous smoking abstinence rate. Low nicotine dependence, high self-efficacy, and combination therapy of varenicline with short-term NRT were associated with a high 6-month continuous abstinence rate. Self-efficacy was a crucial factor for smoking cessation, highlighting that interventions for smoking cessation should focus on increasing self-efficacy. Lastly, our study shows that combination therapy of varenicline with short-term NRT could be a useful option for smokers with intense withdrawal symptoms. As smoking increases the risks associated with COVID-19, a residential smoking cessation program can be an effective alternative in the era of the COVID-19 pandemic.

## Figures and Tables

**Table 1:** Schedules of residential smoking cessation program.

	Day 1	Day 2	Day 3	Day 4	Day 5
08:00		CO ^a^ testing	Breakfast and CO testing		
09:00		Health Check-up (Lab, Chest CT ^b^, Carotid Sono, PFT ^c^, UA ^d^)	Individual counseling <Psychological counseling>	Individual counseling <The result of Health Check-up>	Final individual counseling
10:30		Group Exercise	Recreational session		Closing ceremony
12:00		Lunch			
13:30		Group session <Mindfulness>	Group session <Psychological counseling>	Group Exercise	
14:00	Welcoming Ceremony				
15:00	Psychological assessment	Group session <Behavioral therapy>	Group Exercise	Group session <Behavioral therapy>	
16:00	Group session <Pharmacotherapy>				
17:00	Physician Rounding (Medical counseling and prescription of medications)				
18:00	Dinner				
19:00	Group session <Psychological counseling>	Lecture <Cardiovascular disease and Smoking>	Nutrition management after smoking cessation	Drama therapy	

^a^ CO: carbon monoxide, ^b^ CT: Computed Tomography, ^c^ PFT: pulmonary function test, ^d^ UA: urine analysis.

**Table 2:** Four weeks, 3 months, and 6 months of smoking cessation success (intention-to-treat).

Follow-Up	4 Weeks		3 Months		6 Months	
	Success	Fail	Success	Fail	Success	Fail
Number (%)	552 (96.84)	18 (3.16)	474 (83.16)	96 (16.84)	352 (61.75)	218 (38.25)

Data are presented as the frequency (percentage).

**Table 3:** Characteristics of participants according to 4 weeks, 3 months, and 6 months of smoking cessation success.

Follow-Up	4 Weeks			3 Months			6 Months		
	Success	Fail		Success	Fail		Success	Fail	
	469 (96.90)	15 (3.10)	*p*-Value	396 (81.82)	88 (18.18)	*p*-value	306 (63.22)	178 (36.78)	*p*-Value
Age (yr, mean ± SD)	55.41 ± 10.63	52.53 ± 13.75	0.31	55.85 ± 10.49	52.91 ± 11.56	0.02	55.89 ± 10.20	54.34 ± 11.56	0.13
Gender			0.40			0.03			0.26
M	414 (88.27)	15 (100.00)		357 (90.15)	72 (81.82)		275 (89.87)	154 (86.52)	
F	55 (11.73)	0 (0.00)		39 (9.85)	16 (18.18)		31 (10.13)	24 (13.48)	
Education level (9 data missing)			0.39			0.30			0.17
Middle school or less	51 (11.09)	3 (20.00)		40 (10.31)	14 (16.09)		28 (9.30)	26 (14.94)	
High school	155 (33.70)	3 (20.00)		130 (33.51)	28 (32.18)		101 (33.55)	57 (32.76)	
College or beyond	254 (55.22)	9 (60.00)		218 (56.19)	45 (51.72)		172 (57.14)	91 (52.30)	
Alcohol			0.98			0.56			0.31
Yes	158 (33.69)	5 (33.33)		131 (33.08)	32 (36.36)		98 (32.03)	65 (36.52)	
Regular exercise			0.40			0.24			0.11
Yes	140 (29.85)	6 (40.00)		124 (31.31)	22 (25.00)		100 (32.68)	46 (25.84)	
Hypertension	138 (29.42)	1 (6.67)	0.08	111 (28.03)	28 (31.82)	0.48	86 (28.10)	53 (29.78)	0.70
Diabetes	89 (18.98)	3 (20.00)	0.92	77 (19.44)	15 (17.05)	0.60	63 (20.59)	29 (16.29)	0.25
Dyslipidemia	73 (15.57)	4 (26.67)	0.25	61 (15.40)	16 (18.18)	0.52	50 (16.34)	27 (15.17)	0.73
Any supporters			0.19			0.20			0.23
Yes	401 (85.50)	11 (73.33)		341 (86.11)	71 (80.68)		265 (86.60)	147 (82.58)	
Smoking									
Age of smoking initiation (yr, mean ± SD)	20.61 ± 4.77	18.53 ± 3.25	0.10	20.68 ± 4.65	19.93 ± 5.12	0.18	20.73 ± 4.66	20.23 ± 4.89	0.27
Smoking duration (yr, mean ± SD)	36.20 ± 10.31	35.40 ± 12.74	0.77	36.57 ± 10.28	34.40 ± 10.69	0.08	36.58 ± 9.97	35.48 ± 11.05	0.26
Cigarettes per day (N, mean ± SD)	20.69 ± 8.15	25.33 ± 10.93	0.03	20.33 ± 7.46	23.07 ± 11.00	0.005	20.05 ± 7.52	22.17 ± 9.30	0.006
FTND ^a^ score			0.03			<0.001			<0.001
Mild (1–3)	154 (32.84)	2 (13.33)		140 (35.35)	16 (18.18)		118 (38.56)	38 (21.35)	
Moderate (4–6)	202 (43.07)	5 (33.33)		171 (43.18)	36 (40.91)		121 (39.54)	86 (48.31)	
Severe (7–10)	113 (24.09)	8 (53.33)		85 (21.46)	36 (40.91)		67 (21.90)	54 (30.34)	
Quit attempt in the past year			0.28			0.35			0.51
Yes	172 (36.67)	3 (20.00)		147 (37.12)	28 (31.82)		114 (37.25)	61 (34.27)	
Pharmacotherapy			<0.001			<0.001			0.12
No	12 (2.56)	1 (6.67)		9 (2.27)	4 (4.55)		8 (2.61)	5 (2.81)	
NRT ^b^	158 (33.69)	8 (53.33)		126 (31.82)	40 (45.45)		96 (31.37)	70 (39.33)	
Varenicline	11 (2.35)	4 (26.67)		8 (2.02)	7 (7.95)		7 (2.29)	8 (4.49)	
Varenicline + short-term NRT	288 (61.41)	2 (13.33)		253 (63.89)	37 (42.05)		195 (63.73)	95 (53.37)	
Psychological									
IRC ^c^ rulers (mean ± SD)									
Importance	9.04 ± 1.60	9.13 ± 1.25	0.82	9.03 ± 1.63	9.10 ± 1.41	0.68	9.04 ± 1.60	9.03 ± 1.58	0.95
Readiness	8.26 ± 2.09	7.53 ± 1.96	0.18	8.31 ± 2.05	7.93 ± 2.24	0.12	8.25 ± 2.13	8.23 ± 2.01	0.93
Confidence	7.67 ± 2.17	7.07 ± 2.15	0.29	7.75 ± 2.13	7.17 ± 2.27	0.02	7.80 ± 2.10	7.38 ± 2.27	0.04
Depression scale (CES-D ^d^, 3 data missing)			0.08			0.002			0.37
Normal and Mild depressive symptoms (≤15)	246 (52.79)	5 (33.33)		219 (55.58)	32 (36.78)		166 (54.61)	85 (48.02)	
Moderate depressive symptoms (16~24)	117 (25.11)	3 (20.00)		96 (24.37)	24 (27.59)		73 (24.01)	47 (26.55)	
Severe depressive symptoms (≥25)	103 (22.10)	7 (46.67)		79 (20.05)	31 (35.63)		65 (21.38)	45 (25.42)	
Craving score (TCQ ^e^, mean ± SD)	140.18 ± 40.80	169.14 ± 49.99	0.01	138.99 ± 40.92	150.52 ± 42.26	0.02	140.27 ± 41.73	142.54 ± 40.80	0.58
Self-efficacy scale (mean ± SD)	26.04 ± 6.71	25.07 ± 7.54	0.58	26.34 ± 6.68	24.55 ± 6.81	0.02	26.88 ± 6.63	24.52 ± 6.67	<0.001

Data are presented as the mean ± standard deviation or as the frequency (percentage). ^a^ FTND: Fagerström Test for Nicotine Dependence, ^b^ NRT: nicotine replacement therapy, ^c^ IRC: Importance, Readiness, Confidence, ^d^ CES-D: center for epidemiologic Studies depression scale, ^e^ TCQ: tobacco craving questionnaire.

**Table 4:** Multivariable logistic regression analysis of factors associated with 3 months smoking cessation.

	Model 1 ^a^		Model 2 ^b^		Model 3 ^c^	
	OR (95% CI)	*p*-Value	OR (95% CI)	*p*-Value	OR (95% CI)	*p*-Value
Age (yr, mean ± SD)	1.03s (1.00–1.05)	0.02	1.03 (1.00–1.05)	0.02	1.03 (1.00–1.06)	0.02
Gender						
M	1		1		1	
F	0.49 (0.26–0.93)	0.03	0.49 (0.26–0.93)	0.03	0.61 (0.30–1.24)	0.17
Education level (9 data missing)						
Middle school or less	1		1		1	
High school	1.63 (0.78–3.38)	0.19	2.24 (0.97–5.15)	0.06	1.44 (0.60–3.43)	0.41
College or beyond	1.70 (0.85–3.37)	0.13	2.49 (1.09–5.68)	0.03	1.53 (0.64–3.62)	0.34
FTND ^d^ score						
Mild (1–3)	1		1		1	
Moderate (4–6)	0.54 (0.29–1.02)	0.06	0.56 (0.30–1.07)	0.08	0.52 (0.26–1.02)	0.06
Severe (7–10)	0.27 (0.14–0.52)	<0.001	0.29 (0.15–0.57)	<0.001	0.28 (0.14–0.59)	0.001
Pharmacotherapy						
NRT ^e^	1		1		1	
No	0.71 (0.21–2.44)	0.59	0.67 (0.19–2.31)	0.52	0.80 (0.19–3.36)	0.76
Varenicline	0.36 (0.12–1.06)	0.07	0.36 (0.12–1.07)	0.07	0.39 (0.23–1.26)	0.12
Varenicline + short-term NRT	2.17 (1.32–3.56)	0.002	2.18 (1.32–3.61)	0.002	2.13 (1.25–3.63)	0.006
Depression scale (CES-D ^f^)						
Normal and Mild depressive symptoms (≤15)	1		1		1	
Moderate depressive symptoms (16~24)	0.58 (0.33–1.05)	0.07	0.54 (0.30–0.98)	0.04	0.60 (0.32–1.13)	0.12
Severe depressive symptoms (≥25)	0.37 (0.21–0.65)	0.001	0.36 (0.20–0.65)	0.001	0.48 (0.25–0.91)	0.03
Self-efficacy scale (tertile)						
low	1		1		1	
moderate	1.17 (0.69–1.99)	0.57	0.98 (0.56–1.70)	0.93	0.80 (0.43–1.50)	0.48
high	1.61 (0.89–2.91)	0.12	1.38 (0.75–2.53)	0.30	0.92 (0.46–1.85)	0.82

Data are presented as odds ratios (95% confidence interval), ^a^ Model 1: unadjusted, ^b^ Model 2: adjusted for age and gender, ^c^ Model 3: adjusted for age, gender, education, nicotine dependence (FTND score), pharmacotherapy, Self-efficacy scale, depression (CES-D), ^d^ FTND: Fagerström Test for Nicotine Dependence, ^e^ NRT: nicotine replacement therapy, ^f^ CES-D: center for epidemiologic studies depression scale.

**Table 5:** Multivariable logistic regression analysis of factors associated with 6 months smoking cessation.

	Model 1 ^a^		Model 2 ^b^		Model 3 ^c^	
	OR (95% CI)	*p*-Value	OR (95% CI)	*p*-Value	OR (95% CI)	*p*-Value
Age (yr, mean ± SD)	1.01 (1.00–1.03)	0.13	1.01 (1.00–1.03)	0.13	1.01 (0.99–1.03)	0.26
Gender						
M	1		1		1	
F	0.72 (0.41–1.28)	0.26	0.72 (0.41–1.28)	0.27	0.85 (0.46–1.59)	0.61
Education level (9 data missing)						
Middle school or less	1		1		1	
High school	1.65 (0.88–3.07)	0.12	2.04 (1.03–4.04)	0.04	1.89 (0.91–3.89)	0.09
College or beyond	1.76 (0.97–3.17)	0.06	2.27 (1.15–4.46)	0.02	1.90 (0.93–3.89)	0.08
FTND ^d^ score						
Mild (1–3)	1		1		1	
Moderate (4–6)	0.45 (0.29–0.72)	0.001	0.46 (0.29–0.73)	0.001	0.46 (0.28–0.75)	0.002
Severe (7–10)	0.40 (0.24–0.67)	<0.001	0.42 (0.25–0.70)	0.001	0.46 (0.26–0.81)	0.01
Pharmacotherapy						
NRT ^e^	1		1		1	
No	1.17 (0.37–3.72)	0.79	1.13 (0.35–3.63)	0.83	1.09 (0.29–4.03)	0.90
Varenicline	0.64 (0.22–1.84)	0.41	0.65 (0.22–1.89)	0.43	0.67 (0.22–2.03)	0.48
Varenicline + short-term NRT	1.50 (1.01–2.22)	0.05	1.49 (1.00–2.21)	0.05	1.64 (1.07–2.51)	0.02
Depression scale (CES-D ^f^)						
Normal and Mild depressive symptoms (≤15)	1		1		1	
Moderate depressive symptoms (16~24)	0.80 (0.51–1.25)	0.32	0.76 (0.48–1.20)	0.24	0.86 (0.53–1.40)	0.55
Severe depressive symptoms (≥25)	0.74 (0.47–1.17)	0.20	0.73 (0.45–1.17)	0.19	1.03 (0.61–1.76)	0.90
Self-efficacy scale (tertile)						
low	1		1		1	
moderate	1.10 (0.72–1.69)	0.66	1.03 (0.66–1.59)	0.91	0.95 (0.59–1.54)	0.85
high	2.38 (1.47–3.87)	<0.001	2.24 (1.37–3.68)	0.001	1.97 (1.15–3.37)	0.01

Data are presented as odds ratio (95% confidence interval), ^a^ Model 1: unadjusted, ^b^ Model 2: adjusted for age and gender, ^c^ Model 3: adjusted for age, gender, education, nicotine dependence (FTND score), pharmacotherapy, Self-efficacy scale, depression (CES-D), ^d^ FTND: Fagerström Test for Nicotine Dependence, ^e^ NRT: nicotine replacement therapy, ^f^ CES-D: center for epidemiologic studies depression scale.

**Table 6:** Multivariable logistic regression analysis of factors associated with smoking cessation.

	3 Months Model 3 ^a^		6 Months Model 3 ^a^	
	OR (95% CI)	*p*-Value	OR (95% CI)	*p*-Value
Age (yr, mean ± SD)	1.03 (1.00–1.06)	0.04	1.01 (0.99–1.04)	0.23
Gender				
M	1		1	
F	0.57 (0.28–1.18)	0.13	0.81 (0.43–1.52)	0.51
Education level (9 data missing)				
Middle school or less	1		1	
High school	1.54 (0.62–3.82)	0.36	1.85 (0.88–3.91)	0.11
College or beyond	1.49 (0.60–3.69)	0.39	1.79 (0.85–3.74)	0.12
FTND ^b^ score				
Mild (1–3)	1		1	
Moderate (4–6)	0.50 (0.25–1.02)	0.06	0.46 (0.28–0.76)	0.002
Severe (7–10)	0.33 (0.15–0.71)	0.005	0.52 (0.29–0.93)	0.03
Pharmacotherapy				
NRT ^c^	1		1	
Varenicline + short-term NRT	2.12 (1.24–3.61)	0.006	1.63 (1.07–2.49)	0.02
Depression scale (CES-D ^d^)				
Normal and Mild depressive symptoms (≤15)	1		1	
Moderate depressive symptoms (16~24)	0.55 (0.29–1.06)	0.07	0.80 (0.49–1.31)	0.37
Severe depressive symptoms (≥25)	0.44 (0.23–0.88)	0.02	0.97 (0.56–1.68)	0.92
Self-efficacy scale (tertile)				
low	1		1	
moderate	0.74 (0.39–1.42)	0.37	0.93 (0.57–1.53)	0.79
high	0.79 (0.39–1.62)	0.53	1.75 (1.01–3.03)	0.04

Data are presented as odds ratios (95% confidence interval), ^a^ Model 3: adjusted for age, gender, education, nicotine dependence (FTND score), pharmacotherapy, Self-efficacy scale, depression (CES-D), ^b^ FTND: Fagerström Test for Nicotine Dependence, ^c^ NRT: nicotine replacement therapy, ^d^ CES-D: center for epidemiologic Studies depression scale.

## Data Availability

The data presented in this study are available on request from the corresponding author.
